# Aerodynamics of two parallel bristled wings in low Reynolds number flow

**DOI:** 10.1038/s41598-022-15068-y

**Published:** 2022-06-28

**Authors:** Yu Kai Wu, Yan Peng Liu, Mao Sun

**Affiliations:** Institute of Fluid Mechanics, Beihang University, Beijing, 100191 China

**Keywords:** Fluid dynamics, Biomechanics

## Abstract

Most of the smallest flying insects use bristled wings. It was observed that during the second half of their upstroke, the left and right wings become parallel and close to each other at the back, and move upward at zero angle of attack. In this period, the wings may produce drag (negative vertical force) and side forces which tend to push two wings apart. Here we study the aerodynamic forces and flows of two simplified bristled wings experiencing such a motion, compared with the case of membrane wings (flat-plate wings), to see if there is any advantage in using the bristled wings. The method of computational fluid dynamics is used in the study. The results are as follows. In the motion of two bristled wings, the drag acting on each wing is 40% smaller than the case of a single bristled wing conducting the same motion, and only a very small side force is produced. But in the case of the flat-plate wings, although there is similar drag reduction, the side force on each wing is larger than that of the bristled wing by an order of magnitude (the underlying physical reason is discussed in the paper). Thus, if the smallest insects use membrane wings, their flight muscles need to overcome large side forces in order to maintain the intended motion for less negative lift, whereas using bristled wings do not have this problem. Therefore, the adoption of bristled wings can be beneficial during upward movement of the wings near the end of the upstroke, which may be one reason why most of the smallest insects adopt them.

## Introduction

Tiny insects (with a wing length of about 0.5 mm) are abundant in nature and have significant ecological and biological importance^1–3^. In recent years, an increasing number of researchers have focused their attention on the smallest insects, with a special focus on their flying principles and aerodynamic mechanisms. According to the existing studies on insect morphology, unlike the larger insects with membranous wings, the smallest insects have distinctive bristled wings, e.g., *thrips*^4^, or partially bristled wings, e.g., the tiny wasp *Encarsia Formosa*^5,6^; the specific morphology of the bristled wings can be found in the works of Huber and Noye^5^, Kolomenskiy et al*.*^6^, Kasoju et al*.*^7^ and Zhao et al*.*^8^.

By experimental measurements, Sunada et al*.*^9^ found that the bristled wing could produce the aerodynamic force close to that of the membrane wing, when they made rotational or translational motion at Reynolds number (*Re*) about 10 (*Re* is based on the mean chord-length of the wing and the mean wing-tip speed). He considered this was caused by the formation of virtual barriers between the bristles on the bristle wing. The virtual barriers concept was also adopted by Lee and Kim^10^ to explain why the flow field structures of the bristled wings observed in flow visualization experiments are similar to those of the membrane wings. And they also discussed the effect of Reynolds numbers on the flow field structures of the bristled wings. In order to better characterize the effect of Reynolds numbers on the aerodynamic forces of a bristled wing, Lee et al*.*^11^ proposed a Reynolds number based on the gap width between bristles as the characteristic length, and provided a method for predicting the aerodynamic forces of bristled wings for different characteristic parameters. By carefully analyzing the flow structure of each bristle on the bristled wings, Wu et al*.*^12^ showed that the aerodynamic force production mechanisms of the bristled wings and the membrane wings are different; the aerodynamic force generation of the bristled wings is due to the large surface friction and surface pressure on each bristle in Stokes flow. Recently, Wu et al*.*^13^ showed that when conducting fast acceleration motion, the bristled wing could produce a large aerodynamic force similar to that of a membrane wing; however, unlike the membrane wing that rely on unsteady effect to generate aerodynamic forces, the aerodynamic forces generated by the bristled wing are formed by the superposition of large drag forces produced by each bristle on the quasi-steady Stokes flow.

The bristled wing not only produces aerodynamic forces similar to those of the membrane wing, but also has aerodynamic advantages over the membrane wing in some situations, for example, in the unique “fling” motion of the smallest insects, the drag needed to be overcome when two 2-D bristled wings are separated is significantly reduced compared to the corresponding membrane wings^14^. Similar conclusion can be drawn when generalizing from the two-dimensional case to the there-dimensional one. Experiments indicated that the lift generated by simplified bristled wing models in the “fling” motion at *Re* = 10 is slightly smaller than that of the corresponding membrane wings, but the drag on the bristled wings is greatly reduced. Therefore, the lift-to-drag ratio of the bristled wings is much greater than that of the membrane wings^15,16^. In addition, pausing before the start of fling has been shown to reduce power required.^17^ Moreover, when the ratio of membrane area to the total area of the wings is in the range of *Thrips*’ wings, the bristled wings have the largest lift to drag ratio in the “fling” motion^18^. Recently, a study by Lee et al*.*^19^ found that the bristled wings could alleviate the undesired aerodynamic loading induced by gusty flow.

**Figure 1:**
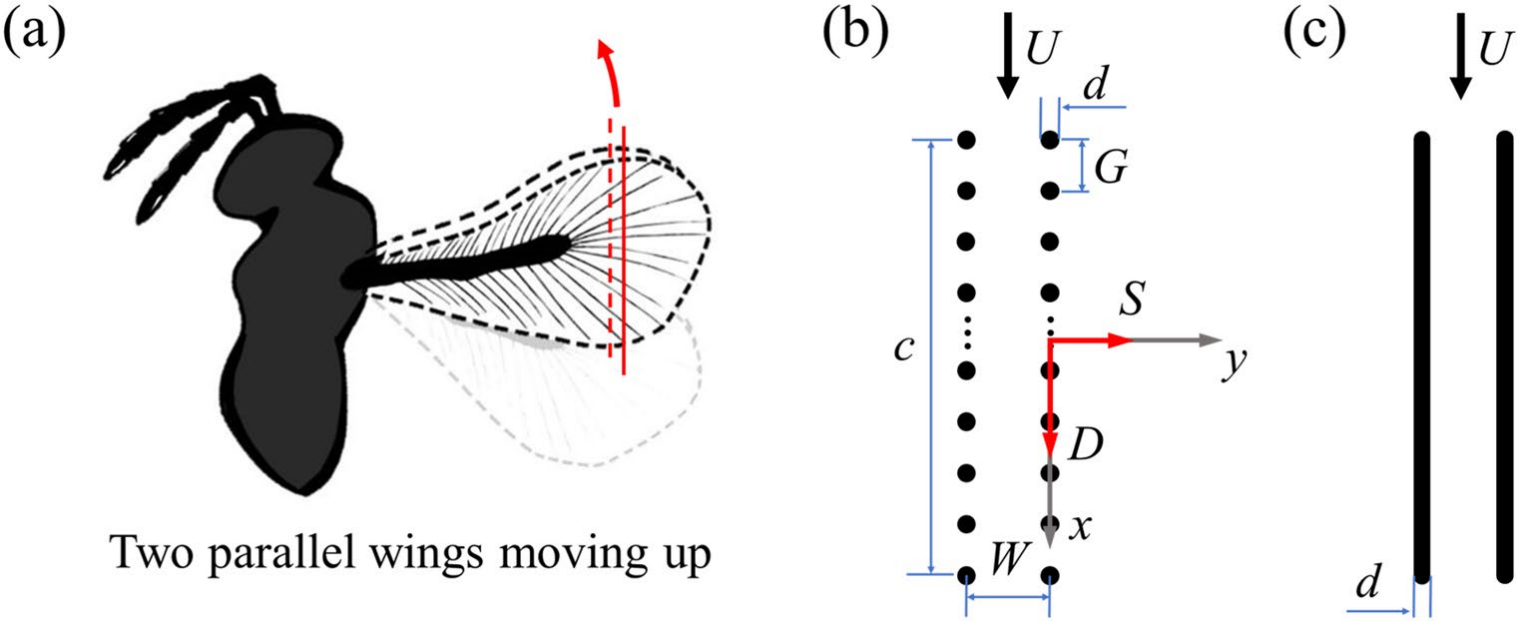
(**a**) Bristle wings are modeled in two dimension using a cross section through the chord of two wings. The red arrow represents the direction of movement of the wings. This diagram was drawn by using the software *Procreate* (version:5.2.5) on the iPad. (**b**) Two bristled wing models at distance of *W* are placed in uniform incoming flow of velocity *U*. (*x*, *y*) are coordinates in a system with its origin at the center of the wing chord *c*. The variables *G* and *d* represent the diameter of the bristle and the distance between two adjacent bristles, respectively. Side force (*S*) and drag (*D*) are the horizontal and vertical component of the aerodynamic force, respectively. (**c**) Two flat-plate wing models with thickness of *d* are placed in uniform incoming flow of velocity *U*.

Recent advances in high-speed photographic technology allow researchers to capture the wings’ motion of smallest insects more accurately and completely during free flight, e.g., experiments on the tiny wasp *Encarsia formosa*^3,20^, *Thrips*^21^ and *Paratuposa placentis*^22^. Based on the measured kinematic data, the flow field and the aerodynamic forces can be obtained by numerical simulation. It is found that when the smallest insect hovering, the vertical force to overcome gravity is mainly produced by the upstroke. During the upstroke, the wing-tip trajectory of the smallest insects is U-shape and the U-shape deepened gradually as the Reynolds number decreased^21^. On the first half of the U-shaped motion, the wings rapidly accelerate downward at a large angle of attack to create a large upward drag contributing most of the total vertical force. This process is called as “rowing” motion^3^. For completing the stroke cycle, the wings need to move upward after the “rowing” motion to prepare for the downstroke, which forms the second half of the U-shape motion, and this process accounts for about 1/4 of the flapping cycle^23^, a rather large percentage of the cycle. Although the angle of attack is zero when the wings move upward, the wings may still produce a large downward aerodynamic drag because of nature of the low Reynolds number flow. Moreover, the upward movement of wings accounts for about a quarter of the flapping cycle, so it is important to study how the smallest insects reduce the generation of negative lift during this process. In previous kinematics studies on the smallest insects, it was found that two wings would be parallel and close to each other at the end of the “rowing” motion, then move upward together at zero angle of attack (as shown in Fig. 1a). Cheng and Sun^23^ found that if two wings are in close proximity when moving upward, the drag on each one of two wings would be reduced compared to an isolated wing, which may attenuate the negative effects of upward wing movement on the flight of the smallest insects. However, they found that during such motion, two membrane wings would produce large horizontal force trying to separate two wings, which may increase the energy consumption in flight.

The above conclusion was obtained by using membrane wings, while the above-mentioned previous studies showed that most of the smallest insects have bristled wings. At present, the aerodynamics of two bristled wings during their upward movement have not been studied. Therefore, it is not known how effective the bristled wings are in reducing drag during upward motion, and whether or not the side force of two bristled wings would be reduced compared to that of the membrane wings. In order to answer the above questions, we use the method of computational fluid dynamics to obtain the aerodynamic forces and flow field pattern generated by two zero-angle-of-attack bristled wings placed in uniform incoming flow parallelly with a small distance in between. For comparison, we also consider the corresponding cases of the flat-plate wings. Furthermore, we analyze the aerodynamic force production mechanisms of these two types of wings as well as the aerodynamic interaction mechanisms between two wings.

## Models and methods

### Wing models and their motions

In a real bristled wing (as sketched in Fig. 1a), on the inner part of the wing, the bristles point in the forward and backward direction, while on the outer part of the wing, the bristles extend mainly along the wing span direction. We selected a cross-section located on the outer part of the bristled wing for simplicity and introduced a pair of simplified two-dimensional bristled wing models, as shown in Fig. 1b. We assumed that they could be a good approximation of real bristled wings; this because for a flapping wing, the outer part of the wing contributes the major part of the total aerodynamic force due to larger velocity there. Furthermore, although the outer part of real wing seems to be highly three-dimensional, the bristles on this part of the wing are very slender (the slenderness ratio, or aspect ratio of a bristle is above 100; see Ref. 9) and extend mainly along the wing span direction. Therefore, it is reasonable to approximate the row of bristles of the real wing by a row of two-dimensional circular cylinders as shown in Fig. 1b. Similar two-dimensional modeling of bristled wings has been used in previous studies^11,14,19^.

As shown in Fig. 1b, the simplified bristled wing model used is composed of *N* equally spaced equal cylinders; the distance between centers of the first and the last cylinders is defined as the chord length *c* of the bristled wing; the diameter of the cylinder is defined as *d*, while the gap between two adjacent cylinders is defined as *G*. According to the real morphology of *Thrips*^9^, the relevant parameters of the bristled wing model are determined as follows: *N* = 21, *d* = 0.5% *c* and *G* = 10*d*, and all parameters are non-dimensionalized by chord length *c*. For comparison, we also consider a two-dimensional flat-plate wing with chord length *c*, whose thickness is the same as the diameter of the cylinder on the bristled wing, and the leading and trailing edges of the flat-plate wing are semi-circles, as shown in Fig. 1c.

As mentioned in the introduction, the upward movement of wings accounts for about a quarter of the flapping cycle. According to the results of kinematics studies^21,23^, two wings are parallel to each other and they moved upward together at a constant velocity; the direction of their motion is parallel to the wing plane (i.e. each of the wings moves in the wing plane), so the angle of attack of the wings is zero. Therefore, our model wings move at constant velocity with zero angle of attack, as shown in Fig. 1b,c.

Based on the previous studies about the smallest insects^1,3,20,21,23^, the Reynolds numbers of both bristled wings and flat-plate wings are 10 in the present study, which are defined as *Re* = *Uc*/*ν*, where *U* is the velocity of the free-stream, *ν* is the kinematic viscosity of the fluid. If the reference length is the diameter of the bristle on bristled wings, the Reynolds number (*Re*_d_) is 0.05. The distance between two wings *W* = 0.1*c*, close to the value of *Thrips*^24^, is mainly considered.

A coordinate system (*x*, *y*) (non-dimensionalized by *c*) is established as shown in Fig. 1b, of which the origin is at the midpoint of the chord of the right wing, while the direction of the *x*-axis and the *y*-axis are parallel and perpendicular to that of the free-stream, respectively. The leading and trailing edges of the wing are located at *x* = − 0.5 and *x* = 0.5 respectively. In the following discussion, the right wing is taken as the representative, since the aerodynamic forces produced by the right and left wings are the same, besides their *y*-components are opposite in direction.

It should be noted that, because of the simplifying assumptions, our study has some limitations. First, the wing model is obtained by taking a cross section of the outer part of the wing and the wing is simplified to a two-dimensional wing. Although the two-dimensional model can reflect the major flow features of the real wings, the three-dimensional effect still needs to be further explored by three-dimensional computation, and our group is planning a three-dimensional study on the flows of bristled wings. Secondly, a flapping wing rotates about the wing root, and a section of the wing moves along a curved line. Our model wing sections move along a straight line (moving upwards); this simplification may cause some error.

### Numerical method

In the present study, numerical simulations are used to obtain the aerodynamic forces on the wings as well as the flow field around them. The governing equations of the flow field are incompressible Navier–Stokes equations, of which the dimensionless form are as follows:

1 $$\nabla \cdot{\mathbf{u}} = 0,$$

2 $${\mathbf{u}}\cdot\nabla {\mathbf{u}} = - \nabla p + \left( {\frac{1}{{\text{Re}}}} \right)\nabla^{2} {\mathbf{u}},$$

where **u** is the non-dimensional fluid velocity, *p* is the non-dimensional fluid pressure, and *Re* is the Reynolds number.

The steady-state solver simpleFoam in OpenFOAM is used to solve Eqs. (1) and (2) with the second-order (Gauss-linear) spatial discretization schemes implemented. SimpleFoam uses the classic SIMPLE (Semi-Implicit Method for Pressure-Linked Equations) algorithm to calculate the coupling of pressure and velocity. Detailed description of the simpleFoam solver can be found in our previous studies^12,13^.

**Figure 2:**
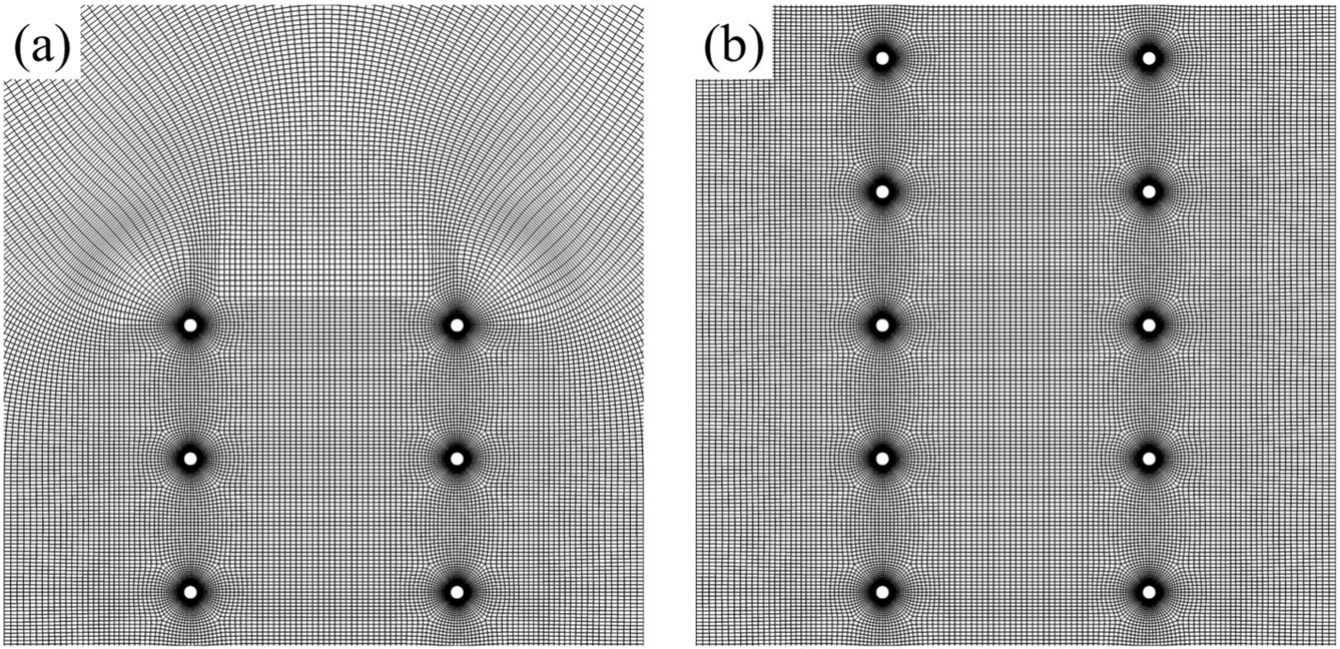
(**a**) A Portion of the grid near the leading edge of two bristled wings and (**b**) a portion of the grid near the middle of two bristled wings.

Structural grids are used in the numerical calculation of two bristled wings (Fig. 2). Similar to the grid of the single bristled wing^12,13^, each cylinder has a body-fitted, O-type sub-grid on the wall. The layer number of the sub-grid is 31, and the thickness of the innermost layer of it is 0.0001*c*. Between adjacent O-type sub-grids of each bristles, there are H-type sub-grids to connect them. A larger H-type sub-grid is used to connect two bristled wings. Outside these regions, there is another large O-type grid, which extends to 60*c* (12 000*d*) from the wing surface; the density of the outer O-type sub-grid gradually decreases as the distance increases. For the grid of two flat-plate wings, there is a body-fitted H-type grid between two flat-plate wings, and an O-type grid with a radius of 60*c* is constructed for the rest of the fluid domain. For comparison, the cases of a single bristled wing and a single flat-plate wing are also calculated, and their grids are the same as those of the previous studies^12,13^. In order to verify the grid-independence of the calculation results, we conducted a grid density test and finally determined the grid cell number of two bristled wings to be about 1 × 10^6^ and the grid cell number of two flat-plate wings to be 6 × 10^5^.

For the boundary conditions, at the far-field inflow boundary, the velocity is specified as the free-stream velocity and the pressure gradient is set to be zero; at the far-field outflow boundary, conditions of zero velocity gradient and free-stream pressure are assigned. On the wing surface, no-slip condition and zero pressure gradient condition are applied.

**Figure 3:**
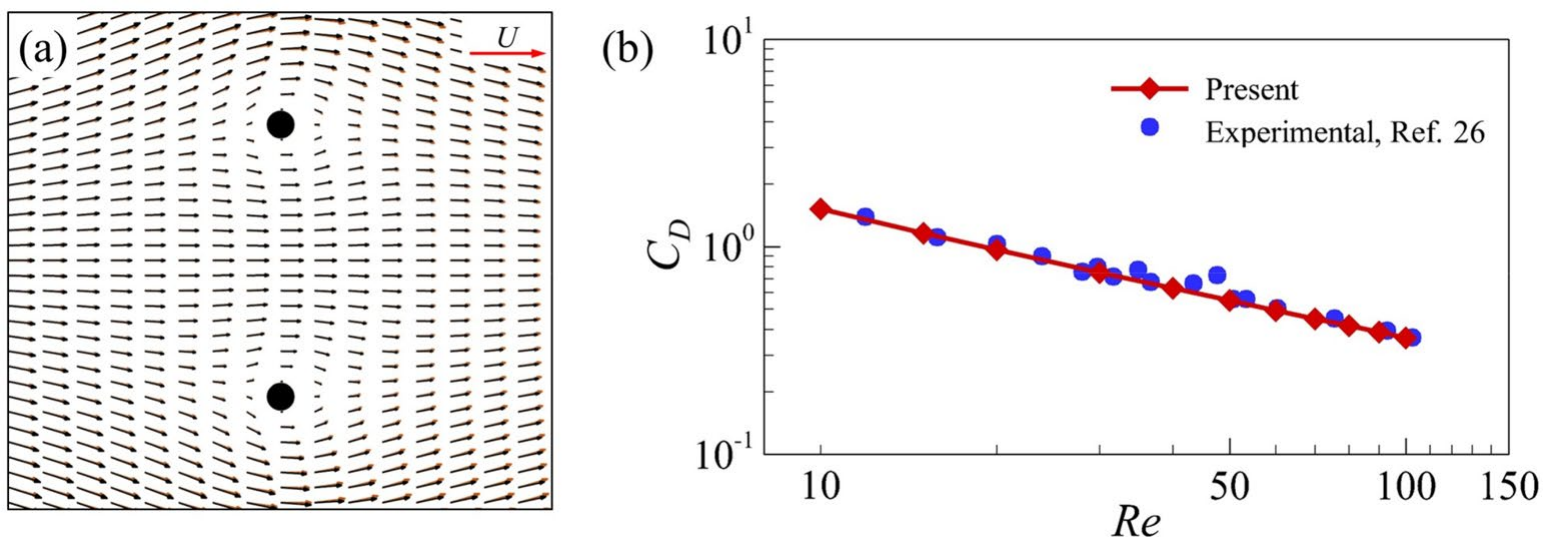
(**a**) Comparison of the velocity vector distribution of two equal cylinders obtained by the present method (black arrow) with the result derived from analytical solution (red arrow). (**b**) Comparison of the drag coefficient of a single flat-plate wing at zero angle of attack obtained by the present method with the experimental result.

The numerical methods used in present study have been validated in previous papers: the drag coefficients of a flat-plate with 90° angle of attack and a single cylinder obtained by this method have been compared with that of previous researches^12,13^; the lift and drag coefficients of two equal cylinders obtained by this method have been compared with that of previous researches as well^12,13^. Here, we conducted further verification using two related cases and compared them with two previous studies^25,26^. In the first case, the flow structure of two equal cylinders in small Reynolds numbers derived from Umemura’s analytical solution^25^ were compared with our results, as shown in Fig. 3a. In this case, the diameter *d* of cylinders is used as the reference length. The distance between the two cylinders is 10*d*, and the Reynolds number (*Re*_d_) is 0.05. In the second one, we compared the experimental measurement results^26^ of the drag coefficient of the zero-angle of attack plate at the Reynolds numbers range of 10 to 100 with our results (Fig. 3b). The agreement seems to be excellent.

## Results and discussion

### Aerodynamic forces on the wings

Since the phase of two wings moving upward together is a special movement during the flight of the smallest insects, which is different from the other part of the flapping motion, the definitions of the aerodynamic forces are also different from the usual ones. In present study, the aerodynamic force in the vertical direction (*x*-direction in Fig. 1) is defined as drag, denoted as *D*, because its direction is the same as that of the incoming flow. The aerodynamic force in the horizontal direction (*y*-direction in Fig. 1) on the wing is defined as the side force, denoted as *S*, because this force points to the outside of two wings and tries to separate the left and right wings. The corresponding force coefficients of drag *D* and side force *S* are *C*_D_ = *D*/(0.5*ρU*^2^*c*) and *C*_S_ = *S*/(0.5*ρU*^2^*c*), respectively, as shown in Table 1 for the single wing and the double wings with *W* = 0.1*c*.

**Table 1:** Aerodynamic force coefficients of wings.

	Single flat-plate wing	One of two flat-plate wings	Single bristled wing	One of two bristled wings
*C*_D_	1.511	0.863	1.490	0.852
*C*_S_	0.000	1.211	0.000	0.145

As shown in Table 1, for the flat-plate wings, the drag coefficient of one of two wings (only 0.863) is 43% lower than that of the single one (about 1.511); while for the bristled wings, one of two wings (only 0.852) is also 43% lower than that of the single one (about 1.490). Comparing the drag coefficients between the flat-plate and bristled wings, it is seen that the latter approximately reaches 99% of the former, whether for both the single wing or each of double wings. Therefore, single wing produces about 1.75 times more drag than one of the double wings regardless of whether they are bristled or flat-plate wings.

As shown in the Table 1, the side force of each one of the two flat-plate wings is 1.211, which is larger than that of the bristled wings (0.145) by about an order of magnitude. During flight, the insects need to make an effort to overcome the side forces for keeping the two wings from separating from each other. Thus, if the smallest insects use membrane wings, their flight muscles need to overcome very large side forces, whereas using bristled wings does not have this problem. Therefore, bristled wings can be beneficial during upward movement, which may be one reason why most of the smallest insects adopt them.

In summary, for the bristled wings, each one of two wings generates less drag forces than the single wing; the same is true for the flat-plate wings. Furthermore, the side force acting on each one of two bristled wings is smaller than that of the flat-plate wings by nearly an order of magnitude.

### Aerodynamic force production mechanisms

**Figure 4:**
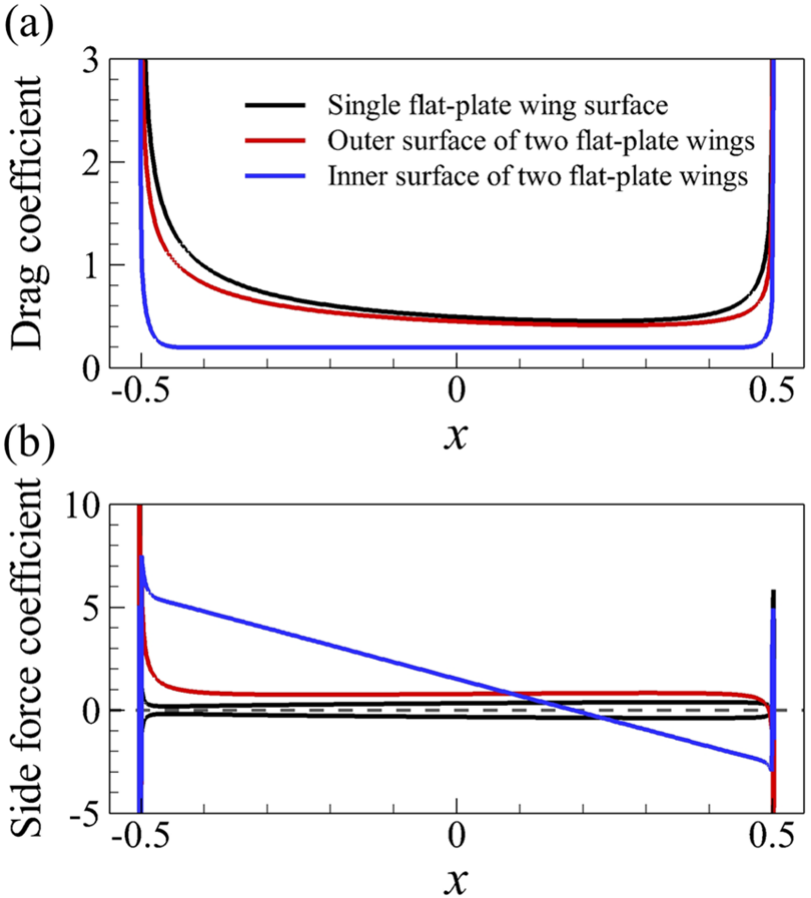
(**a**) The drag coefficient distribution on the single flat-plat wing and one of two flat-plate wings. (**b**) The side force coefficient distribution on one of two flat-plate wings.

First, we examine the cases of the flat-plate wings. Figure 4 shows the force coefficient on the flat-plate wing surfaces. Since the drag coefficient distribution on the upper and lower surface of the single flat-plate wing are the same, only that of one surface is given. As can be seen from the figure, the main reason why the drag of one of two flat-plate wings is greatly reduced compared to that of the single one is that the drag coefficient on the inner wing surface is reduced. For the side force, the outer surface of one of two flat-plate wings contributes positively; the most of inner surface has large positive contributions to the side force, except for the last 30% of the inner surface.

In order to facilitate the following discussion of the contribution of surface pressure and surface friction to the aerodynamic coefficient, we define the surface pressure coefficient and non-dimensional shear stress as *C*_*p*_ = (*p*—*p*_∞_)/(0.5*ρ*U^2^) and *C*_*f*_ = *τ*/(0.5*ρ*U^2^), respectively, where *τ* is the shear stress on the body surface.

**Figure 5:**
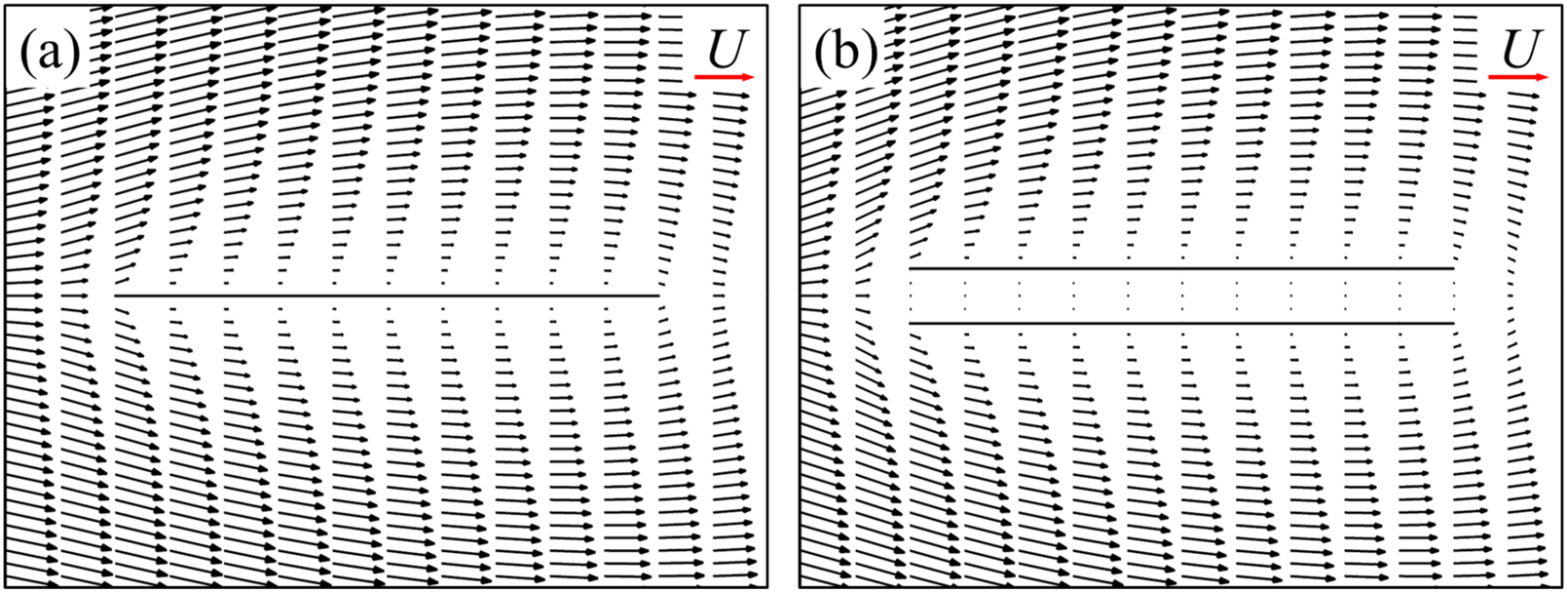
The plot of non-dimensional velocity vector around (**a**) a single flat-plate wing and (**b**) two flat-plate wings.

As is widely known, for the single flat-plate wing parallel to the free-stream, the drag acting on the wing is mainly contributed by surface friction. The integral calculation of the surface pressure and wall shear stress on the single flat-plate wing indicates that the integral result of the surface pressure coefficient (*C*_*p*_) in *x*-direction is 0.074, accounting about 5% of the total drag, while the corresponding value of non-dimensional shear stress (*C*_*f*_) is 1.437, accounting about 95%. The large viscous friction produced on the wing surface is due to the viscous shear layers formed near the wall of the flat-plate wing (Fig. 5a). For a pair of flat-plate wings, the integral results of the surface pressure coefficient (*C*_*p*_) and the non-dimensional shear stress (*C*_*f*_) in *x*-direction is 0.043 and 0.820, respectively. The integral results of non-dimensional shear stress on the inner surface of one of two flat-plate wings is 0.211, which is 70.5% lower than that of the single flat-plate wing (0.7185). This explains the results in Fig. 4a. Comparing the flow fields of a single flat-plate wing and those of two flat-plate wings (Fig. 5a,b), it is seen that due to the aerodynamic interaction of two flat-plate wings, the velocity of the fluid relative to the inner surface of two flat-plate wings is very small. Thus, the corresponding reduced velocity gradient of the fluid in *y*-direction weaken the drag on both wings substantially, which explains the significant decrease of the surface friction on the inner surface of two flat-plate wings compared to that of the single flat-plate wing. However, due to the small flow-interference effect on the outer surface (comparing Fig. 5a,b), the drag coefficient distribution on the outer surface of two flat-plate wings is similar to that on the surface of single flat-plate wing, as shown in Fig. 4a.

**Figure 6:**
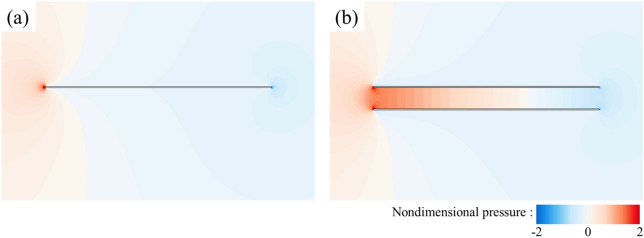
Pressure contour plots of (**a**) a single flat-plate wing and (**b**) two flat-plate wings.

**Figure 7:**
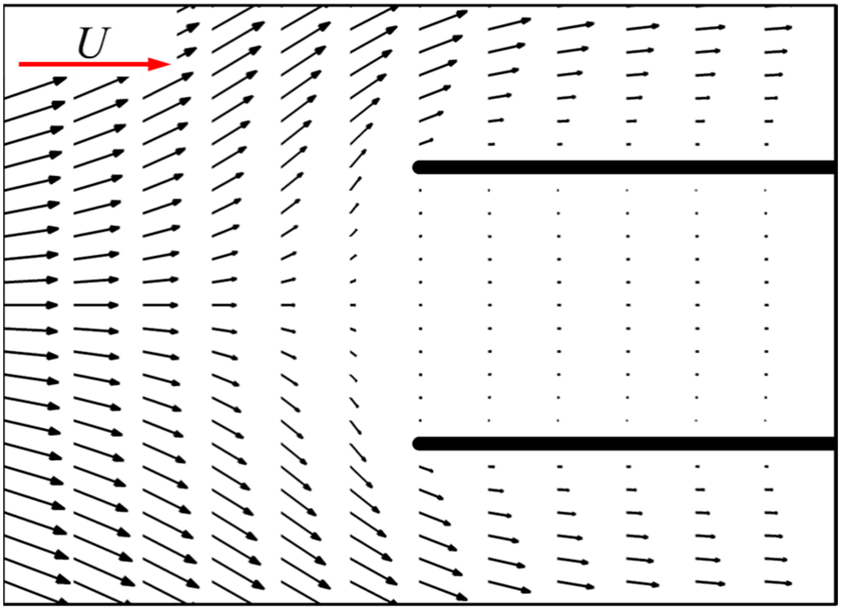
The plot of non-dimensional velocity vectors around the leading edges of two flat-plate wings [first 15% of the two flat-plate wings shown in Fig. 5b].

Now, let us focus on the side force production mechanisms of the flat-plate wings. From the results in the previous section (Table 1), it can be seen that one of two flat-plate wings would produce large side force (1.211), which even exceeds the drag coefficient of it. By integrating the surface pressure coefficient (*C*_*p*_) in *y*-direction, it can be obtained that the integral value is 1.194, which contributes about 99% of the side force (1.211). Comparing Fig. 6a,b, it is obvious that the pressure contour distribution near the outer surface of two flat-plate wings is similar to that of the single flat-plate wing. Therefore, the side force is mainly produced by the change in fluid pressure near the inner surface of two flat-plate wings. As shown in Fig. 6b, the fluid pressure on the inner side of two flat-plate wings is larger than that on the outer side, so there is a large pressure difference between the inner and outer sides of two flat-plate wings, resulting in large side forces. The reasons for the formation of the pressure difference can be elucidated by the flow field distribution shown in Fig. 7. The fluid velocity towards the inner side of two flat-plate wings decreases rapidly in front of the inlet due to the interaction between the wings which reduces the velocity on the inner side and obstruct the flow through the middle gap between two wings. As the fluid velocity decreases sharply over a short distance, the viscous dissipative effect is relatively small, while most of the kinetic energy of the fluid is converted into fluid potential energy (static pressure). Therefore, a large positive pressure is formed near the leading edges of two flat-plate wings (Fig. 6b). In the current study, due to the small Reynolds number, the flow is dominated by viscous effects, which gradually dissipate the potential energy of the fluid, leading to the decrease of the static pressure along the middle gap between two wings. Separating at the leading edge and flowing through the outer side of two wings, the fluid converges again at the trailing edge and induce negative pressure around the trailing edge. The above analysis explains the reduction of friction and the generation of large side force of two flat-plate wings.

**Figure 8:**
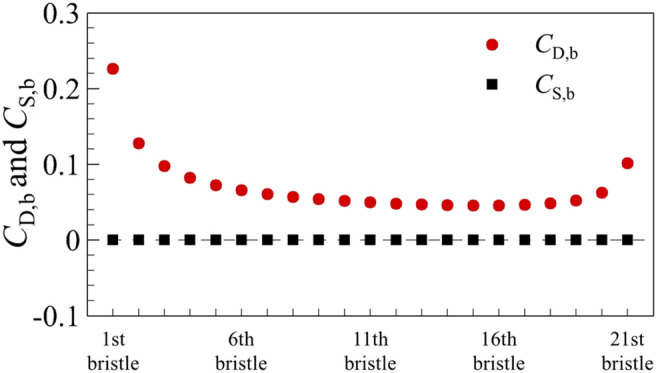
The drag coefficient (*C*_D,b_) and side force coefficient (*C*_S,b_) on each bristle of a single bristled wing.

Next, the cases of bristled wings are considered. The case of a single bristled wing will be discussed first. The aerodynamic force of bristled wing is jointly contributed by the bristles on it. The drag coefficient and the side force coefficient of bristles are written as *C*_D,b_ and *C*_S,b_, respectively (for convenient comparison, the chord length is still used as the characteristic length of *C*_D,b_ and *C*_S,b_). Figure 8 plots *C*_D,b_ and *C*_S,b_ of each bristle on a single bristled wing. Counting from the leading edge, the drag coefficient of the first bristle is the highest, and it goes down in order until the fifth, and the difference between the adjacent bristles also decrease gradually, while from the sixth to eighth, it remains almost constant. However, from the nineteenth near the trailing edge, the drag coefficient of each bristle gradually increases again with the increment much smaller than that of the bristles near the leading edge. Since the flow of each bristle is symmetrical about *x* direction, there is no side force on each bristle (Fig. 8).

**Figure 9:**
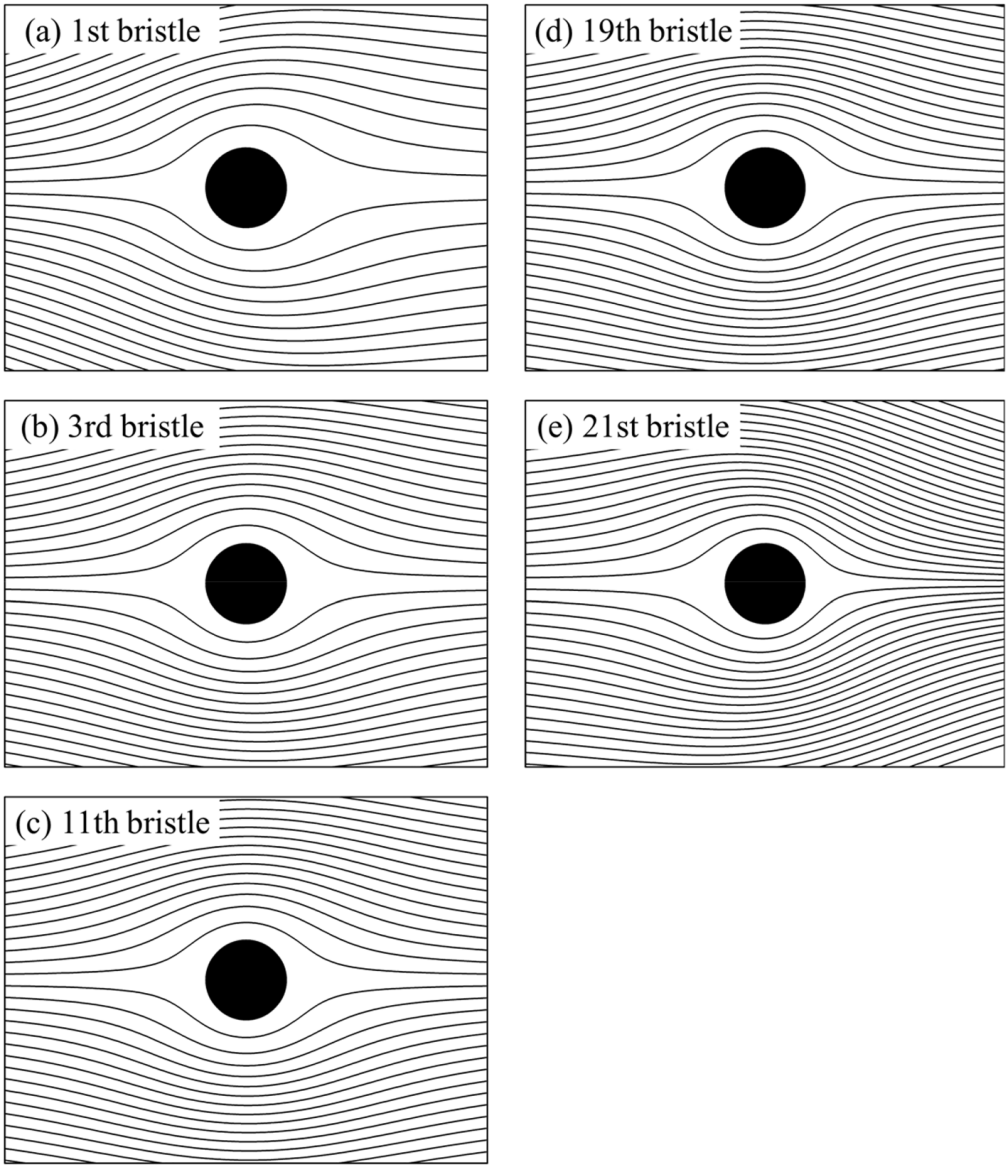
Streamline pattern of (**a**) the first, (**b**) the third, (**c**) the eleventh, (**d**) the nineteenth, and (**e**) the twenty-first bristle of a single bristled wing.

In the present study, at Reynolds number Re = 10 where the characteristic length is the wing chord length, corresponding to the bristled Reynolds number *Re*_d_ = 0.05 where the characteristic length is the bristle diameter *d*, the streamline patterns of the partial bristles are shown Fig. 9. Except that the streamline distribution of the first bristle and the 21st bristle is no longer left–right symmetric (Fig. 9a, e), the streamline distribution of the remaining bristles is approximately left–right symmetric. Moreover, the integral calculation of the surface pressure and shear stress on the single bristled wing indicates that the integral result of the surface pressure coefficient (*C*_*p*_) in *x*-direction is 0.736, accounting about 49.4% of the total drag, while the corresponding value of non-dimensional shear stress (*C*_*f*_) is 0.754, accounting about 50.6%, which shows that the contributions of the surface pressure and surface friction on drag are nearly equal. The integral calculation of each bristle surface shows that the contributions of the surface pressure and surface friction on drag of each bristle are also nearly equal. Therefore, the flow around each bristle is a typical Stokes flow (analytical solutions of creeping flows around a circular cylinder and a sphere showed that the pressure and frictional drags contribute to the total drag of the cylinder on the ratio 1:1, and for the sphere, the ratio is 1:2^27^). The aerodynamic force coefficients generated by bristles in Stokes flow are very high, and add up to produce large aerodynamic forces on the bristled wing. However, because of the interaction of bristles, the aerodynamic forces on them are substantially reduced to be comparable with a single bristle. Due to the different degree of interactions, the aerodynamic forces of bristles are different (Fig. 8). We will discuss the mechanisms of interaction of bristles on the bristled wing in detail below.

**Figure 10:**
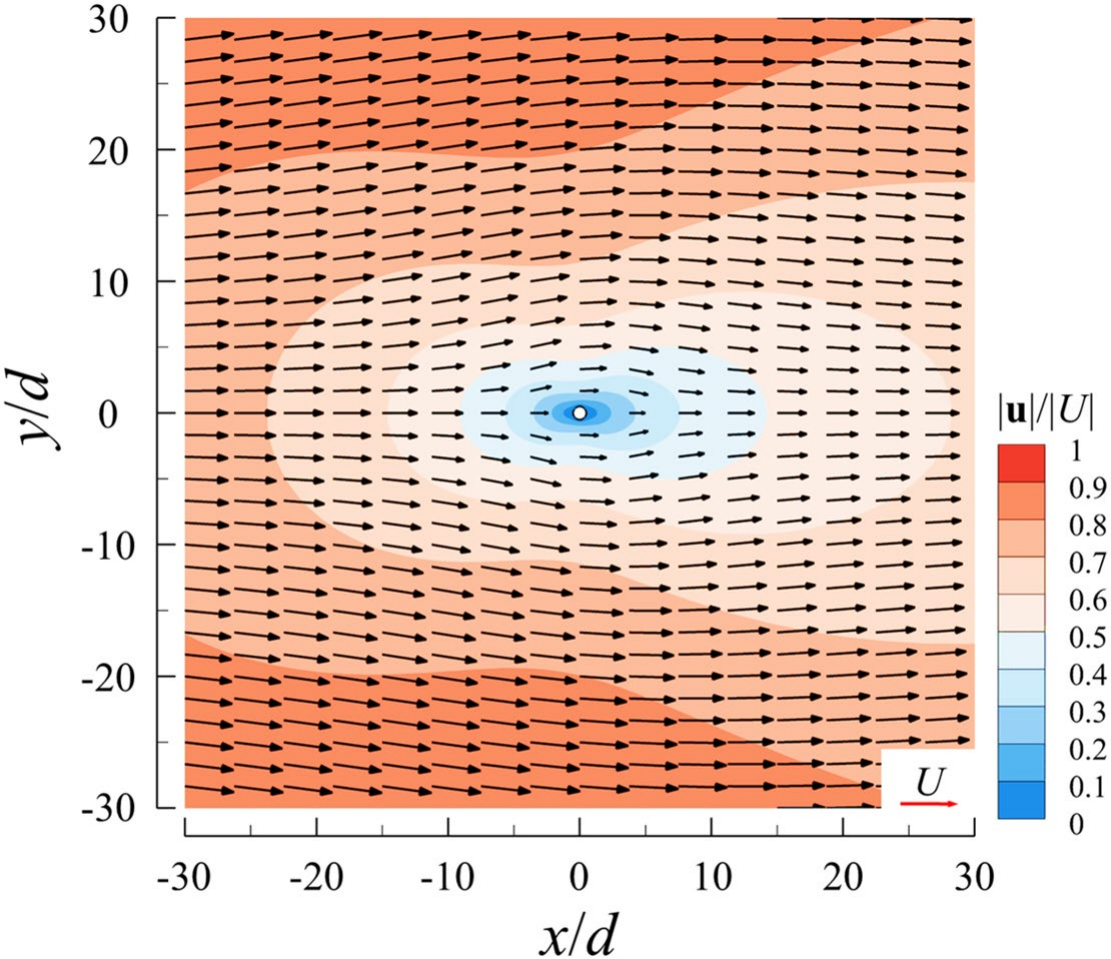
The plot of non-dimensional velocity vector and its magnitude contours around an isolated cylinder.

To explain the above mechanisms of interaction between the bristles of a single bristled wing, we first examine the flow field around an isolated cylinder (bristle). Figure 10 gives the distribution of velocity magnitudes around an isolated cylinder (bristle) with *Re*_d_ = 0.05. It is seen that in the direction perpendicular to the free-stream, an isolated cylinder could reduce the flow velocity significantly, even for locations far away from it, *e.g.*, the velocity at 20*d* from the center of the cylinder reduces to 80% of the free-stream velocity; the range of such influence in the upstream direction is even wide, can extend 30*d*; the downstream direction is the most severely affected area, in which the velocity of the fluid located at 30*d* from the center is reduced to only about 60% of the free-stream velocity. In small Reynolds number flow (*Re*_d_ = 0.05), there would be a thick viscous shear layer formed around an isolated cylinder. According to the description of the bristled wing model above, the distance between adjacent bristles is only 10*d*, thus it is not difficult to find that the viscous shear layer of each bristle would cover several adjacent bristles and affect their flow.

**Figure 11:**
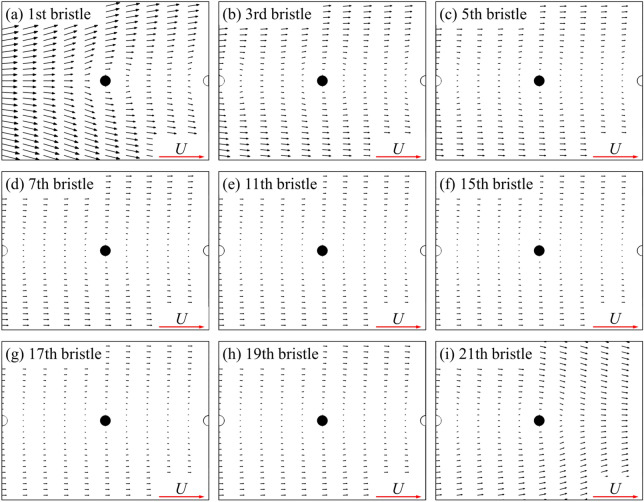
The plot of non-dimensional velocity vector around (**a**) the first, (**b**) the third, (**c**) the fifth, (**d**) the seventh, (**e**) the eleventh, (**f**) the fifteenth, (**g**) the seventeenth, (**h**) the nineteenth and (**i**) the twenty-first bristle of a single bristled wing.

Knowing how the shear layer of an isolated cylinder (bristle) affects the surrounding fluid, we could explain the mechanisms of interaction among the bristles on a single bristled wing. For the first bristle on the leading edge of a single bristled wing, it is only influenced by downstream bristles. Since an isolated cylinder (bristle) has limited range of perturbation of the fluid in the upstream direction, the first bristle is only affected by 3–4 downstream bristles. Therefore, the flow around this bristle is least affected, as shown in Fig. 11, which explain why the drag coefficient of the first bristle is the highest. For the 2nd to 5th bristles (as shown in Fig. 11b,c), the disturbance influence on these bristles gradually increases, due to the increasing number of bristles in the upstream direction. Therefore, drag coefficients of these bristles gradually decrease as the number of bristles in the upstream direction increase. However, the influence of a single cylinder (bristle) on the surrounding fluid is finite. Therefore, when the number of upstream bristles increases beyond a certain value, the impact of such modification is strongly limited. However, the impact of the downstream bristles stays the same, resulting in the unchanged velocity distribution around the bristles in the middle of the wing (as shown in Fig. 11d–g). This explains why the variation in the drag coefficient from the 6th bristle to the 18th bristle is very limited. However, from 19th to the 21st bristle, as the number of the downstream bristles is reduced, the influence from the downstream gradually decreases, and the velocity around these bristles increases again (as shown in Fig. 11h,i), which makes the surface pressure and frictional forces of these bristles increase gradually. Therefore, the drag generated by the bristled wing parallel to the free-stream is similar to that of the flat-plate wing, but their drag production mechanisms is distinctive different.

**Figure 12:**
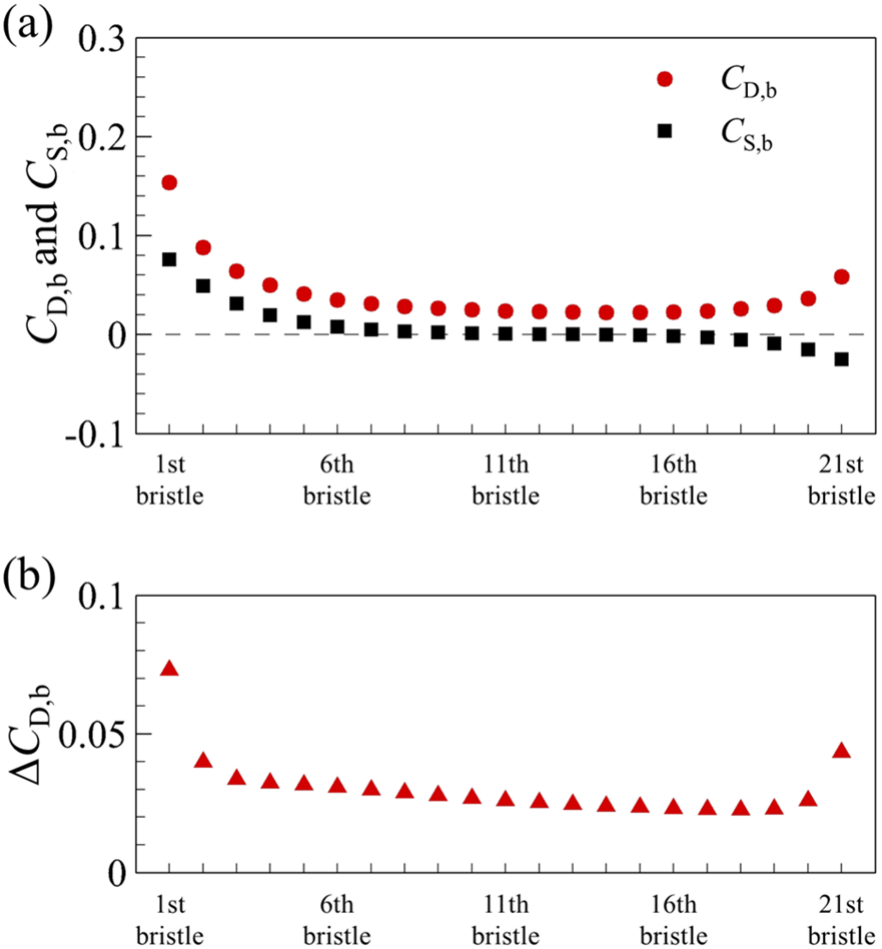
(**a**) The drag coefficient (*C*_D,b_) and side force coefficient (*C*_S,b_) on each bristle of the upper one of the two bristled wings. (**b**) The relative change in *C*_D,b_ on each bristle (Δ*C*_D,b_) between single bristled wing and the right wing of two bristled wings.

Next, we examine the case of two bristled wings. From the above (Table 1), the drag coefficient of each of two bristled wings is significantly reduced compared to that of a single bristled wing. Since the aerodynamic forces of the bristled wing is a superposition of that of each bristle, we need to examine the drag and side force coefficients of each bristle as well. Because the aerodynamic forces and flow field structure of two bristled wings are symmetrical to each other, we focus on upper one of two bristled wings (i.e., right wing) in the following content. The integral calculation of the surface pressure and wall shear stress on one of two bristled wing indicates that the integral result of the surface pressure coefficient in *x*-direction is 0.422, accounting about 49.5% of the total drag, while the corresponding value of non-dimensional shear stress is 0.430, accounting about 50.5%, which shows that the contributions of the surface pressure and surface friction on the drag is equal. The same is true for the integral calculation of each bristle surface separately. Figure 12a shows the drag coefficient and side force coefficient of each bristle on one of two bristled wings. Compared with the case of single bristled wing (Fig. 8), the drag coefficient on each bristle is reduced, which results in a consequent reduction in the total drag of the bristled wings. The relative change on each bristle is defined asΔ*C*_D,b_, as shown in Fig. 12b. In addition, different from the fact that there is no side force produced by each bristle of a single bristled wing, the bristles near the leading and trailing edges of each one of two bristled wings would generate side forces which is perpendicular to the free-stream. As shown in Fig. 12a, the largest side force, approximately 0.076, is generated by first (leading edge) bristle and points to the positive *y* direction. The side forces gradually decrease in order from the 2nd to 5th bristles gradually, and approaches zero among the 6th to 17th bristles. However, from the 18th to 21th ones located near the trailing edge, the side forces increase again from 0 to 0.025, which points to the negative *y* direction. Therefore, the main reason for the small side force of each one of two bristled wings is the small number of bristles with non-zero side force, unlike the flat-plate wings on which the side force coefficient everywhere is not zero (Fig. 4b).

**Figure 13:**
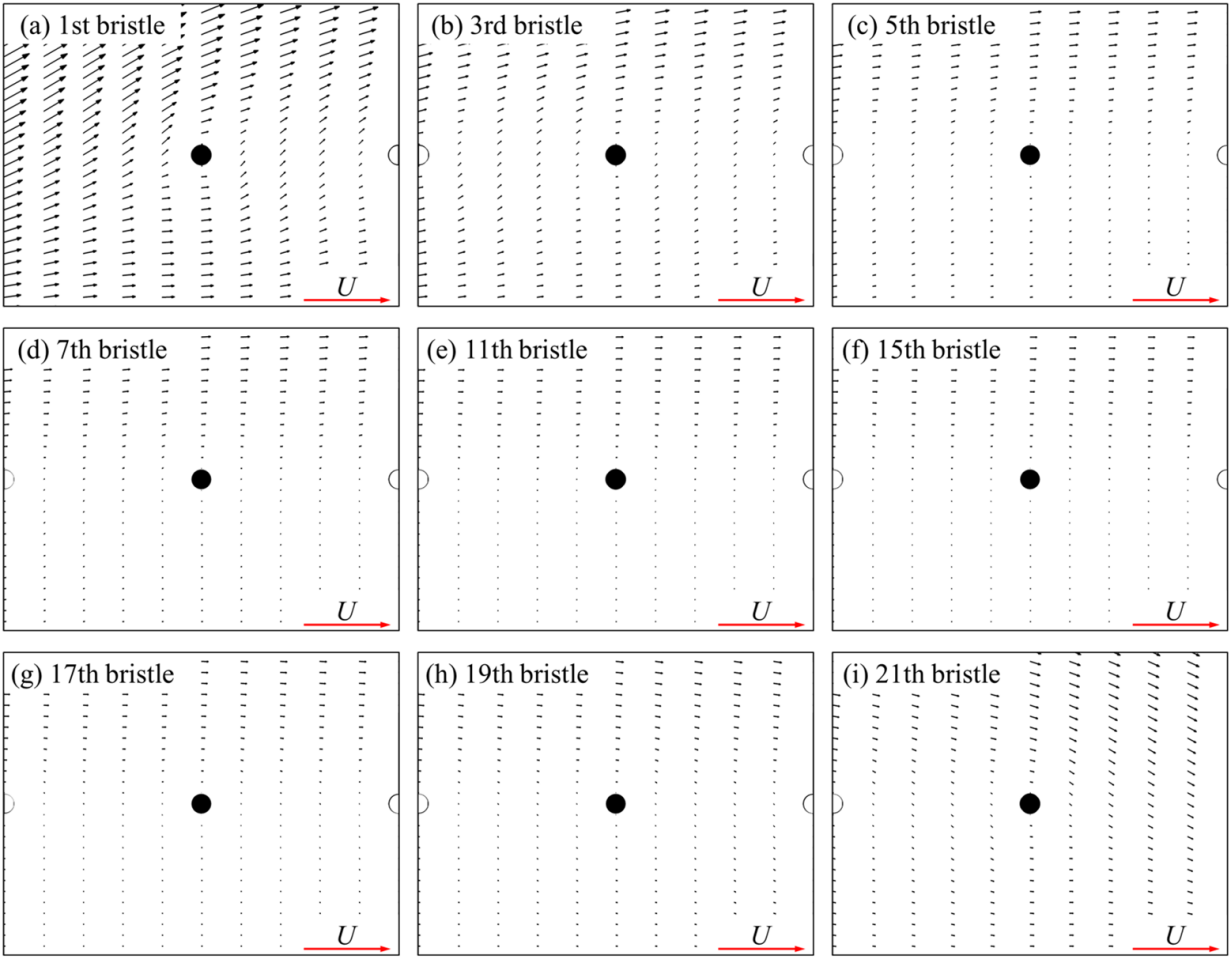
The plot of non-dimensional velocity vector around (**a**) the first, (**b**) the third, (**c**) the fifth, (**d**) the seventh, (**e**) the eleventh, (**f**) the fifteenth, (**g**) the seventeenth, (**h**) the nineteenth and (**i**) the twenty-first bristle of the upper one of two bristled wings.

As shown in Fig. 13, the velocity around each bristle of two bristled wings is significantly smaller than that of a single bristled wing (Fig. 11), due to the mutual perturbation of the viscous layers of two bristled wings, leading to the reduction of surface pressure and frictional forces on each bristle. Therefore, the drag reduction mechanisms of two bristled wings is different from that of two flat-plate wings, which is clearly seen from the proportions of surface pressure and friction contributions to the drag of the two types of wings.

**Figure 14:**
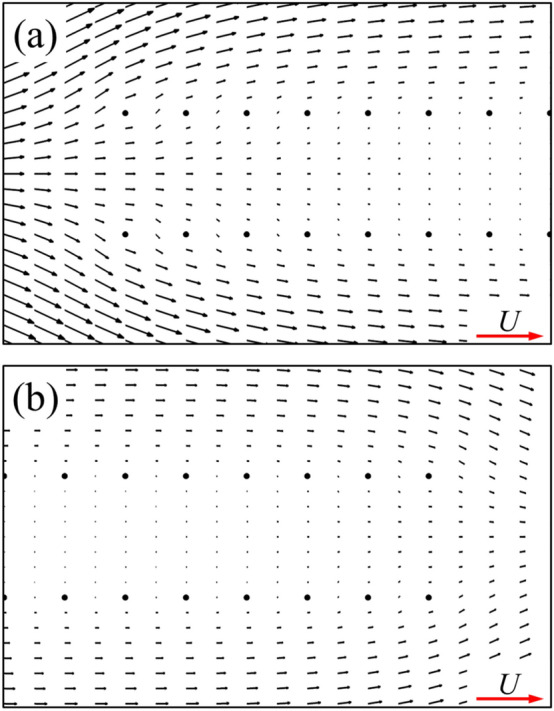
The plot of non-dimensional velocity vector around (**a**) the leading edge and (**b**) the trailing edge of two bristled wings.

**Figure 15:**
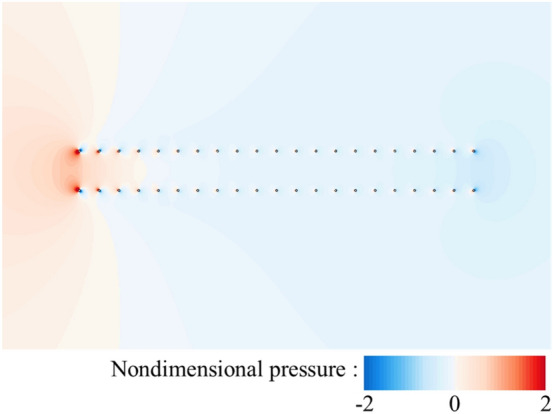
Pressure contour plots of two bristled wings.

Next, let us consider the side force production mechanisms of two bristled wings. Figure 14 shows the velocity vector distribution around the bristles near the leading edges of two bristled wings. If we set the upper one of two bristled wings (right wing) in this figure as an example, the flow direction of the fluid around the bristles near the leading edge is deflected counterclockwise relative to the free-stream direction, due to the pressure difference between the inside and outside of the two bristled wings (Fig. 15), which is caused by the interaction of two bristled wings. There is leakage flow through the gap between the bristles near the leading edge, and by integrating the non-dimensional velocity in *y*-direction, it can be obtained that the leakage flow is 0.0039. Therefore, the aerodynamic forces of these bristles are deflected, generating an aerodynamic component in the positive *y* direction. The flow field of bristles near the trailing edge of two bristled wings is shown in Fig. 14b. Again, choosing the upper wing as an example, the mutual influence of two bristled wings leads to the clockwise deflection of the flow velocity direction of several bristles near the trailing edge relative to the free-stream direction, resulting in a negative *y*-axis side force. As seen from the discussion here, the mechanisms for generating side forces in the bristled wings is completely different from that in the flat-plate wings.

In conclusion, the drag of the flat-plate wing is contributed by the surface friction generated by the viscous shear layer. Due to the fluid velocity in the middle of two flat-plate wings decrease dramatically, which leads to a significant reduction in the friction force on the inner wing surface, the drag of each one of two flat-plate wings is smaller than that of the single flat-plate wing. Furthermore, two flat-plate wings also produce side forces of equal value and opposite directions, which are caused by a large pressure difference between the inner and outer wing surfaces. The bristled wing generates almost the same drag as the flat-plate wing, but the drag production mechanisms is different. The drag force on a bristled wing is a superposition of the large surface pressure and the surface friction generated by each bristle in the Stokes flow. When the two bristled wings are placed in parallel, the drag of each one is also reduced, because the flow velocity around them decreases due to the interaction of bristles on two bristled wings. The interaction of bristles on two bristled wings also deflects the flow velocity direction of several bristles located near the leading and trailing edges, so that side forces of equal value and opposite directions are generated on two bristled wings. However, only a few bristles produce side force actually; and the side forces produced by the bristles near the leading and trailing edges are in opposite directions, so cancel each other out partially. Therefore, the side force generated by one of two bristled wings is smaller than that of one of two flat-plate wings. We only consider the aerodynamic mechanism during the upward movement of the bristled wings rather than that of the entire flapping cycle, so the conclusions here are only applicable to this motion phase.

### The effect of the distance between two wings on the aerodynamic-forces

In the above sections, the non-dimensional distance between two wings (*W*/*c*) remains constant at 0.1. This is only an approximation based on the data of real wings of *Thrips* and other tiny insects^3,23^. Therefore, it is necessary to examine the drag and side force coefficients of two wings with different distances in between. In addition to the original case (*W*/*c* = 0.1), we considered the case of two wings with a smaller distance (*W*/*c* = 0.05) in between and a larger distance (*W*/*c* = 0.2) in between. As above, the Reynolds number *Re* = 10 was chosen for the simulations and the same dimensionless approach was used for the aerodynamic forces.

Table 2 summarizes drag coefficients (*C*_D_) and side force coefficients (*C*_S_) of one of two flat-plate wings and one of two bristled wings with different distances in between (*W*/*c*). As shown in the table, when the distance of two wings decreases, the *C*_D_ of the flat-plate wing decreases, while the *C*_S_ gradually increases. For the bristled wings, the *C*_D_ has similar trend as that of the flat-plate wing, but the side force coefficient decreases gradually in the range considered.

**Table 2:** Aerodynamic force coefficients of wings with different wing distances.

	*W*/*c* = 0.05	*W*/*c* = 0.1	*W*/*c* = 0.2
**One of two flat-plate wings**			
*C*_D_	0.813	0.863	0.945
*C*_S_	1.428	1.211	1.103
**One of two bristled wings**			
*C*_D_	0.806	0.852	0.938
*C*_S_	0.063	0.145	0.361

**Figure 16:**
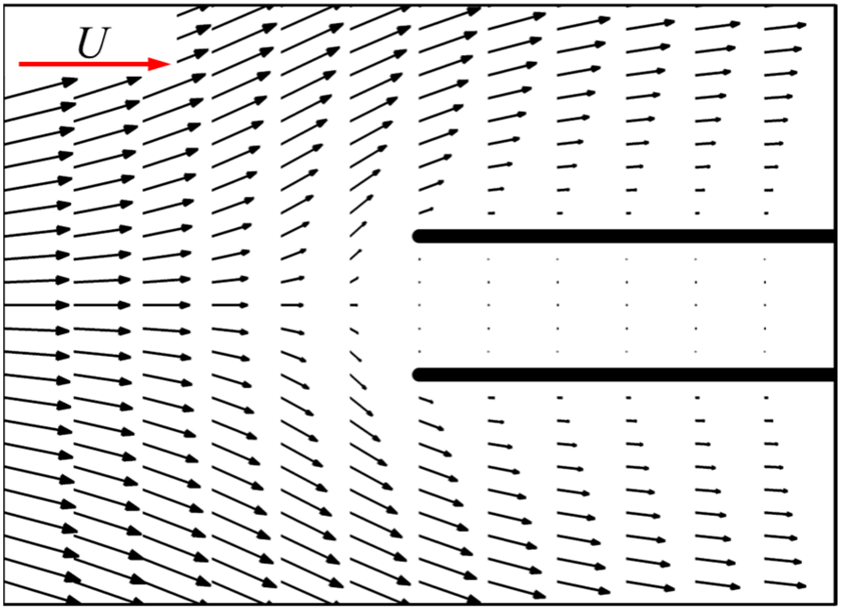
The plot of non-dimensional velocity vector around the leading edge of two flat-plate wings with a smaller distance in between (*W*/*c* = 0.05).

**Figure 17:**
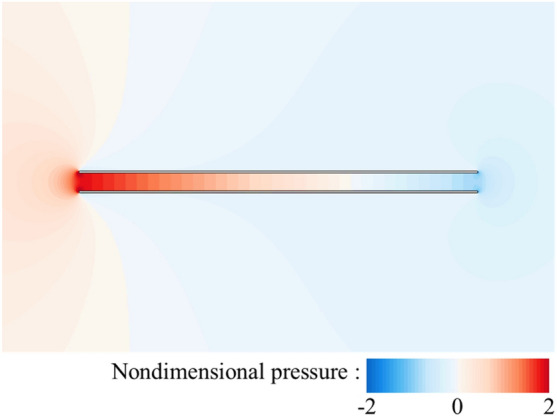
Pressure contour plots of two flat-plate wings with a smaller distance in between (*W*/*c* = 0.05).

For two flat-plate wings, as the distance between them decreases, the obstruction effect on internal flow of two flat-plate wings is enhanced, leading to the decrease of the velocity of the internal fluid (comparing Figs. 7 and 16) and the reduction of the surface friction on the inner wing surface. Moreover, the decrease in the distance between two flat-plate wings also results in a sharper change in fluid velocity near the leading edge of two flat-plate wings (comparing Figs. 7 and 16). Therefore, the fluid pressure in this area is greater (comparing Figs. 6 and 17), leading to a greater side force.

**Figure 18:**
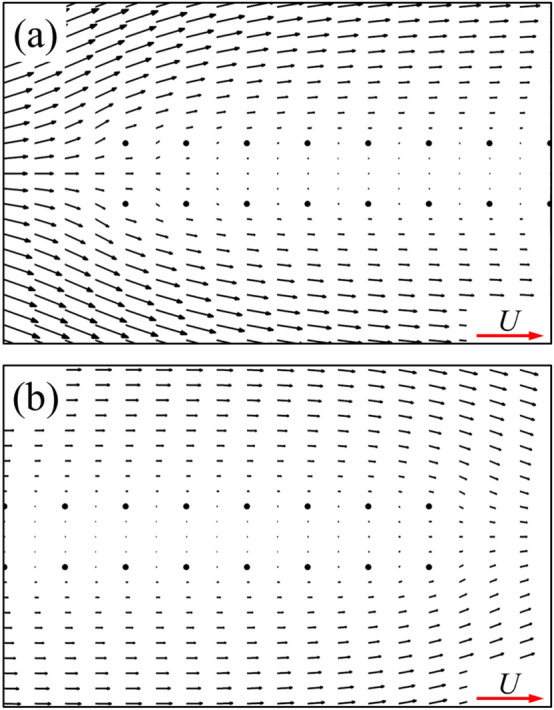
The plot of non-dimensional velocity vector around (**a**) the leading edge and (**b**) the trailing edge of two bristled wings with a smaller distance in between (*W*/*c* = 0.05).

For two bristled wings, when the wing distance *W* decreases, the interaction between the bristles on two bristled wings is enhanced, leading to the decrease in the flow velocity of each bristle, and thus the surface pressure and frictional force acting on each bristle reduce as well. However, the side force decreases with the decreasing distance, which is opposite to the changing tendency of the side forces on flat-plate wings. As above, the side force of the bristled wings is generated by the deflection of each bristle’s flow velocity. When the wing distance decreases, the range of fluid, in which the velocity direction is deflected, narrows down (comparing Figs. 14 and 18). In other words, the leakage flow through the gaps occurs only between the first to the third bristle. By integrating the non-dimensional velocity in *y*-direction, it can be obtained that the leakage flow is 0.0013 which is much smaller than that of the case when *W*/*c* = 0.1. Thus, the number of bristles generating side forces is reduced and the side force on the bristled wings are consequently reduced. Similarly, when the wing distance increases, the number of bristles affected by the deflection of the fluid velocity increases, and the side force on the bristled wings increases as well.

## Conclusion

In the present study, the aerodynamic forces and their production mechanisms of two bristled wings (placed parallelly in uniform incoming flow at zero-angle-of-attack with a very small distance in between) are investigated by numerically solving the N-S equations. For comparison, we also consider the corresponding cases of the flat-plate wings. The results are as follows. The drag on each one of two bristled wings is decreased 40% compared to that of a single wing; the same is true for the flat-plate wings. However, the drag reduction mechanisms of these two types of wings are different. For two bristled wings, due to the interaction two wings, the velocity around each bristle is reduced compared to the case of a single bristled wing, and thus the surface pressure and frictional force of each bristle are reduced. For two flat-plate wings, due to the interaction between two wings, the fluid velocity on the inner side of two wings are significantly slowed down, leading to the reduction of friction on the inner wing surface. When two wings are placed in uniform incoming flow parallelly, only a very small side force act on each bristled wing, but the side force acting on each flat-plate wing is much larger than that of each bristled wing (larger by nearly an order of magnitude). This is due to the different production mechanisms of side forces in two cases. The results presented here suggest that the utilization of bristled wings can greatly reduce the side force that the muscles must overcome, compared to that of membrane wings. Therefore, the adoption of bristled wings can be beneficial during upward movement of the wings near the end of the upstroke, which may be one reason why most of the smallest insects adopt them.

## Data Availability

The data that support the findings of this study are available from the corresponding author upon request.
